# Spatial correlation network structure of operational efficiency and its driving factors: A case study of star-rated hotels in China

**DOI:** 10.1371/journal.pone.0313500

**Published:** 2024-11-14

**Authors:** Jilei Tao, Xiulong Jin, Hai Cheng, Qinan Wang

**Affiliations:** 1 School of Geographic Information and Tourism, Chuzhou University, Chuzhou, China; 2 Graduate School, Southwest Forestry University, Kunming, China; University of Messina: Universita degli Studi di Messina, ITALY

## Abstract

Accurately grasping the spatial network correlation structure of operational efficiency in star-rated hotels and its influencing factors is of great significance for promoting high-quality coordinated development of star-rated hotels and the regional tourism reception industry. Using a comprehensive approach integrating the Super-DEA model, modified gravity model, and social network analysis to explore the evolving characteristics of spatial network structure in operational efficiency of provincial star-rated hotels in China from 2013 to 2022, and their underlying mechanisms. The results indicate that: (1) During the study period, the average operational efficiency of star-rated hotels in China was 0.618, with spatial efficiency values showing a distribution pattern of "Eastern > Central > Western > Northeastern". (2) The spatial correlation network of operational efficiency among provincial star-rated hotels in China overall exhibits characteristics of densification, hierarchical structure, and reinforcement. Provinces such as Beijing, Tianjin, Shanghai, and Jiangsu in the eastern region play central roles as "central actors" in the network, while provinces in the western and northeastern regions such as Heilongjiang, Tibet, and Xinjiang play roles as "passive actors" in the spatial network. (3) Members of the “Net benefit” and “Two-way spillover” are primarily from the eastern and some central provinces, while members of the “Net overflow” are mainly from the northeastern and western provinces. (4) Factors such as economic development level, residents’ consumption level, and distance from provincial capital cities collectively drive the evolution and optimization of the spatial network structure of operational efficiency among provincial star-rated hotels in China. This study not only enriches the research findings on hotel operational efficiency but also provides a reference for constructing a cross-regional collaborative mechanism to enhance the operational efficiency of star-rated hotels.

## 1. Introduction

Tourism has emerged as a strategic pillar industry in China’s national economy and social development. The Opinions of the State Council on Promoting the Reform and Development of the Tourism Industry underscore the industry’s advancement through transformation, upgrading, and enhancing quality and efficiency. Star-rated hotels are accommodation facilities primarily focused on lodging services, as assessed by the Chinese tourism administrative departments, and they are equipped with functions for business, conferences, leisure, and vacations [1]. As an integral part of the tourism reception industry, star-rated hotels directly influence the enhancement of regional tourism industry efficiency through their operational efficiency [2, 3]. Due to the differences in the natural environment and the level of socio-economic development in various regions of China, the development level of the star-rated hotel industry is uneven, and there is a large gap in operational efficiency [4]. To strengthen regional collaboration and improve overall operational efficiency, it is necessary to understand the spatial and temporal dynamic evolution of the operational efficiency of star-rated hotels, grasp the spatial correlation network of the operational efficiency of star-rated hotels from the global perspective, and explore the spatial correlation characteristics and the formation mechanism of the spillover effect.

Operational efficiency is the best basis for evaluating a hotel’s operations and has always been of great interest. In the 1980s, some scholars, supported by evidence, highlighted the risks associated with low productivity in the hotel industry [5, 6]. They began to draw lessons from studies on manufacturing productivity, discussing how to assess hotel productivity from the perspectives of inputs and outputs [7], and also proposed recommendations to enhance hotel productivity from a management technology standpoint [8]. In the 1990s, research on hotel production efficiency was further expanded. Michael Baker and Michael Riley compared the concepts of hotel productivity and manufacturing productivity, clarifying the characteristics of hotel productivity [9]. Nick Johns et al. used Data Envelopment Analysis (DEA) to monitor the productivity of 15 chain hotels over the course of a year, comparing differences between different quarters [10]. Anderson et al. assessed the management efficiency level in the hotel industry using Stochastic Frontier Analysis (SFA) [11]. In the 21st century, the number of research findings on hotel efficiency has continuously increased, which is crucial for optimizing hotel resource allocation and enhancing core competitiveness [12]. In numerous studies, methods for measuring hotel operational efficiency primarily include two types: Data Envelopment Analysis (DEA) [13, 14] and Stochastic Frontier Analysis (SFA) [15, 16]. The study subjects mainly include chain hotels [17, 18] and star-rated hotels [4, 19]. The main contents of the research include measurement of hotel production efficiency [20], analysis of factors influencing hotel production efficiency [16, 21], and strategies to enhance hotel production efficiency [22]. The spatial scope of the research covers various levels, specifically including hotel operational efficiency at global [23], national [24], provincial [25], and city [26] scales. In the selection of hotel operational efficiency indicators, scholars tend to include factors such as the number of hotels, their star ratings, the number of employees, fixed asset investments, etc., as input indicators [16, 18, 27]. Output indicators typically include revenue, room occupancy rate, and business taxes [14, 23, 25].

Scholars have conducted extensive research on the operational efficiency of hotels, providing significant reference value for industry development. However, with the gradual formation of a networked transportation system and continuous development of internet information technology, coupled with the implementation of regional economic integration policies, the depth and breadth of economic development cooperation between regions have been continuously enhanced, leading to increasingly closer spatial connections among various factors of production [28]. The limitations of previous research findings are becoming increasingly apparent. On one hand, there is a lack of holistic research methods that overlook the impact of non-adjacent regions on the operational efficiency of hotels. On the other hand, there is a tendency to focus on analyzing attribute data while not giving enough importance to relational data. This approach only reflects the current state of hotel operational efficiency in various regions and fails to outline the complex spatial association hierarchy and nested relationships between regional hotel operational efficiencies within the context of a networked society. This makes it difficult to identify and implement targeted measures to improve the overall efficiency of the hotel industry in a region. Social Network Analysis (SNA), as an interdisciplinary analytical method [29], enables comprehensive analysis of spatial correlation networks and their structures within a system using "relationship data." It effectively overcomes the limitations of "attribute data" [30] and has been widely applied in recent years to research complex relational networks across various fields. However, few scholars have applied it to studies of hotel economic development.

**Fig 1:**
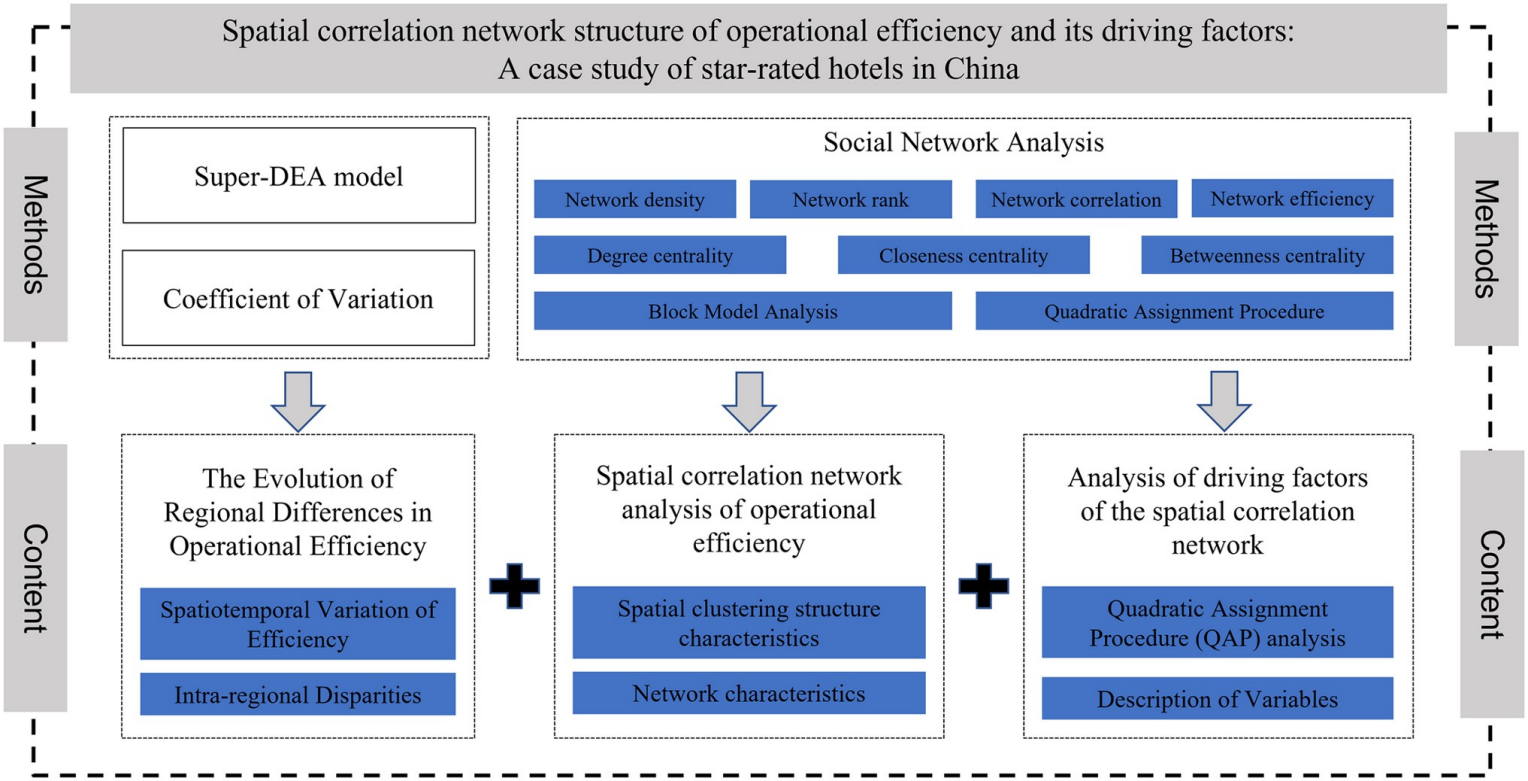
Article content structure flowchart.

Therefore, this study employs the Super-DEA model to calculate the operational efficiency of star-rated hotels in 31 provinces and cities of China from 2013 to 2022 (excluding data from Hong Kong, Macau, and Taiwan). Data Envelopment Analysis (DEA), compared to the Stochastic Frontier Analysis (SFA), avoids the influence of subjective factors and does not require data standardization. It has an absolute advantage in dealing with problems involving multiple inputs and outputs [31]. The Super-DEA model, an improvement over the traditional DEA model, not only distinguishes efficient units from an efficiency value perspective but also considers the projection issues of efficient units, making it more practical and effective in management decision analysis applications [32]. Therefore, the Super-DEA model was selected for the calculation of efficiency in star-rated hotels. Building on this, the study utilizes a modified gravity model and SNA methods to construct the spatial correlation network of operational efficiency among star-rated hotels across provinces in China. This approach aims to reveal both the overall and individual network structure characteristics, clarify the positions of different regions within the network structure, and employ the Quadratic Assignment Procedure (QAP) method to identify factors influencing the spatial correlation network of operational efficiency among star-rated hotels in China. The goal is to provide insights that contribute to fostering coordinated regional hotel industry development and enhancing industry efficiency. The specific structure of the article is shown in Fig 1.

## 2. Research methods and data sources

### 2.1. Super-DEA model

The DEA method, known as Data Envelopment Analysis, is a performance evaluation method developed based on the concept of relative efficiency. It constructs an optimal frontier based on analyzing input-output indicators to assess the relative effectiveness of decision-making units [33]. The Super-DEA model to provide more detailed information compared to the traditional DEA method, assisting decision-makers in better understanding the strengths of efficient units and potential areas for improvement, thereby making better decisions [34]. For the hotel industry, its controllability of inputs relative to outputs is advantageous. To facilitate horizontal comparisons of operational efficiency among star-rated hotels in different regions, the study adopts an input-output approach. It employs the Super-DEA model to calculate hotel operational efficiency. The specific formula is as follows:

$$\begin{array}{l} \text{min}\theta \\ S.T.\left\{\begin{array}{l} \sum_{j=1}^{n}\lambda_{j}X_{j}+S^{-}=\theta X_{i},i=1,2,3\ldots \ldots ,n\\ \sum_{j=1}^{n}\lambda_{j}Y_{j}-S^{+}=Yi,i=1,2,3\ldots \ldots ,n\\ \lambda_{j}\geq 0,j=1,2,3\ldots \ldots ,n\\ S^{+}\geq 0,S^{-}\geq 0 \end{array}\right. \end{array}$$ (1)

In this formula, *θ* represents the operational efficiency of hotels, with a value range of 0 < *θ* ≤ 1. A larger *θ* indicates higher operational efficiency in the region, while a smaller *θ* suggests lower operational efficiency. *X*_*j*_ and *X*_*i*_ represent the input factor *j* and the input factor of region *i*, respectively; *Y*_*j*_ and *Y*_*i*_ represent the output factor *j* and the output factor of region *i*, respectively; *S*^*-*^ and *S*^*+*^ are slack variables, representing excess input and insufficient input, respectively; *λ*_*j*_ is the weight variable of the decision-making unit (DMU).

### 2.2. Coefficient of variation

The coefficient of variation is an effective method for measuring the dispersion of a dataset [35]. It is used to evaluate the degree of variation in operational efficiency among star-rated hotels within different regions of China. The specific formula is as follows:

$$CV=\frac{\sum_{i=1}^{n}{\left(y_{i}-\bar{y}\right)}^{2}/n}{\bar{y}}$$ (2)

In this formula, *CV* represents the coefficient of variation. A larger *CV* indicates greater differences in operational efficiency among star-rated hotels within the region, while a smaller *CV* suggests lesser differences. *y*_*i*_ represents the operational efficiency of star-rated hotels in province *i*; $\bar{y}$ is the average operational efficiency of star-rated hotels in the entire region; and *n* denotes the number of provinces within the region.

### 2.3. Modified gravity model

The construction of spatial correlation matrices is the basis for the application of social network theory and methodology. The gravity model can effectively reflect spatial variations in a certain attribute’s values due to geographic distance, economic development levels, and changes in population distribution. It quantitatively measures the spatial correlation strength of production efficiency [36] and is an effective method for constructing spatial correlation matrices. Therefore, introducing a modified gravity model that considers economic geographic distance, using Gross Domestic Product (GDP) to calculate gravity coefficients, constructs a spatial correlation matrix for the efficiency of operating star-rated hotels. The specific formula is as follows:

$$F_{ij}=K_{ij}\frac{N_{i}N_{j}}{{D_{ij}}^{2}},K_{ij}=\frac{G_{i}}{G_{i}+G_{j}},{D_{ij}}^{2}={\left(\frac{d_{ij}}{g_{i}-g_{j}}\right)}^{2}$$ (3)

In the formula, *F*_*ij*_ represents the operational efficiency linkage strength of star-rated hotels in various regions of China; *K*_*ij*_ denotes the gravity coefficient; *N* stands for the operational efficiency of star-rated hotels in each region; *D*_*ij*_ signifies the economic geographic distance; *d*_*ij*_ denotes the spatial distance between provincial capital cities in different regions; *G* and *g* respectively denote the GDP and per capita GDP of each region. To facilitate the exploration of the overall and individual network structural characteristics of operational efficiency in star-rated hotels in China, the mean value of each row’s data in the matrix is used as a threshold for binary processing. If the linkage strength is greater than the threshold, assign a value of 1, indicating the presence of operational efficiency linkage between provinces; otherwise, assign a value of 0, indicating no linkage.

### 2.4. Social network analysis

#### 2.4.1. Overall and individual network characteristic indicators

Indicators such as network density, network rank, and network correlation reflect the overall network characteristics of operational efficiency in star-rated hotels [37]. Degree centrality, betweenness centrality, and closeness centrality indicate the position and role of various regions in China’s operational efficiency network of star-rated hotels [38]. Specific indicators are introduced in Table 1.

**Table 1:** Characteristic indicators of star-rated hotel operational efficiency network.

Indicator	Formulas	Variable meaning
Network density	*D* = *M*/[*N*(*N* − 1)]	*D* denote network density as a measure of the completeness of overall network relationships. *M* represent the actual number of connections, and *N* represent the number of nodes within the network.
Network rank	*H* = 1 − *K*/max(*K*)	*H* denote network rank as a measure of asymmetry in the accessibility between nodes within the network. *K* denote the number of node pairs, max(*K*) denote the maximum possible number of node pairs.
Network correlation	*C* = 1 − 2*V*/[*N*(*N* − 1)]	*C* represent the network correlation, which serves to measure the robustness of the network structure. *V* denote the number of mutually inaccessible node pairs, and *N* represent the total number of nodes within the network.
Network efficiency	*E* = 1 − *M*/max(*M*)	*E* represent network efficiency, which quantifies the connectivity efficiency between provinces within the network. *M* denote the number of redundant lines in the network, and max(*M*) represent the maximum possible number of redundant lines.
Degree centrality	*DC* = *n*/(*N* − 1)	*DC* denote the degree centrality, a measure of the attractiveness and connectivity of a node with respect to others. *N* represent the total number of nodes in the network, and *n* signify the actual number of connections a particular node has within the network.
Closeness centrality	$CC=\left(N-1\right)/\sum_{j=1}^{n}d_{ji}$	*CC* represent closeness centrality, indicating the degree of independence of a node within the spatial network. *d*_*ij*_ denote the shortest distance between province *i* and province *j*.
Betweenness Centrality	$BC=\frac{2\sum_{j=1}^{n}\sum_{k=1}^{n}G_{jk}\left(i\right)}{N^{2}-3N+2}$	*BC* denote betweenness centrality, a measure of the extent to which a node acts as a bridge connecting other provinces. *G*_*jk*_ represent the number of shortest paths between provinces *j* and *k* and n be the number of provinces.

#### 2.4.2. Block model analysis

Block Model is a method for exploring the patterns of network clustering structures [39]. It groups nodes based on the characteristics of their connections, thereby revealing the structural features and association patterns among nodes in the network. This paper draws on the methodology developed by Wasserman and Faust for evaluating trends in intra-block relationships within networks, analyzing the role of various positions in the development of regional elements [40]. If block *B*_*k*_ contains *g*_*k*_ nodes, then the total number of possible relationships within *B*_*k*_ is given by the combination formula *g*_*k*_×(*g*_*k*_−1); If the network in which module *B*_*k*_ is situated contains a total of *G* nodes, then the potential number of relationships between module *B*_*k*_ and the entire network is *g*_*k*_×(*G*−1); The expected proportion of total relationships for module *B*_*k*_ is (*g*_*k*_-1)/(*G*-1). Based on this metric, which evaluates the relationships both within and between positions, modules can be categorized into four distinct attribute characteristics, as illustrated in Table 2.

**Table 2:** Classification criteria for spatial correlation network plates.

The actual internal relationship ratio within the tectonic plate	The proportion of relationships received by the tectonic plate	
	≈0	>0
⩾ (*g*_*k*_ − 1)/(*G* − 1)	Two-way spillover plate	Net benefit plate
⩾ (*g*_*k*_ − 1)/(*G* − 1)	Net overflow plate	Agent plate

#### 2.4.3. Quadratic Assignment Procedure

The Quadratic Assignment Procedure (QAP) is a non-parametric method encompassing both QAP correlation analysis and QAP regression analysis [41]. It assumes no prior independence among independent variables, thus adeptly avoiding the pitfalls of multicollinearity often encountered in traditional analyses when variables are presumed to be highly correlated within theoretical frameworks [42]. QAP correlation analysis is based on the permutation of matrices, deriving a correlation coefficient by comparing the similarity of values at corresponding positions between matrices, and then conducting a non-parametric test. QAP regression analysis is used to examine the regression relationships between multiple independent variable matrices and a dependent variable matrix, and to evaluate the significance of the determination coefficient *R*^2^. Through Quadratic Assignment Procedure (QAP) to investigate the key factors affecting the spatial correlation intensity of operational efficiency in China’s star-rated hotels.

### 2.5. Data sources and selection of indicators

Based on data availability and completeness, the study primarily selected sample data from 31 provinces and municipalities in China from 2013 to 2022 (excluding Hong Kong, Macau, and Taiwan) as the research subjects. The study is conducted at the provincial level for several reasons. Firstly, the number of provinces in China is appropriate for conducting a nationwide relational network analysis. If the study scale were set at the city level, the multitude of network relationships would be too vast, potentially hindering effective analysis and visualization. Secondly, the national tourism authorities only provide standardized statistics for star-rated hotels at the provincial level, making it impossible to obtain data at the city level. The number, classification, and personnel of star-rated hotels are sourced from the official website of the Ministry of Culture and Tourism of the People’s Republic of China (https://zwgk.mct.gov.cn/). The regional GDP, per capita GDP, resident consumption levels, total import and export volumes, and other social statistical data are sourced from the China Statistical Yearbook. Tourist arrivals data are sourced from the China Tourism Statistical Yearbook and the statistical bulletins on National Economic and Social Development published by various provinces and municipalities. The spatial distances between provincial capitals were calculated using ArcGIS.

The scientifically reasonable input-output indicators are the foundation and key for evaluating the operational efficiency of star-rated hotels. This article draws on existing evaluation systems, adhering to principles such as systematic, scientific indicator selection, and data availability. It also considers the matching between the number of units in the input-output model and the number of input-output indicators. In the end, the selection of input indicators primarily focuses on capital and labor, specifically including the number of hotels, original value of fixed assets, and number of employees. Output indicators primarily focus on revenue, selecting the operating income of star-rated hotels.

**Fig 2:**
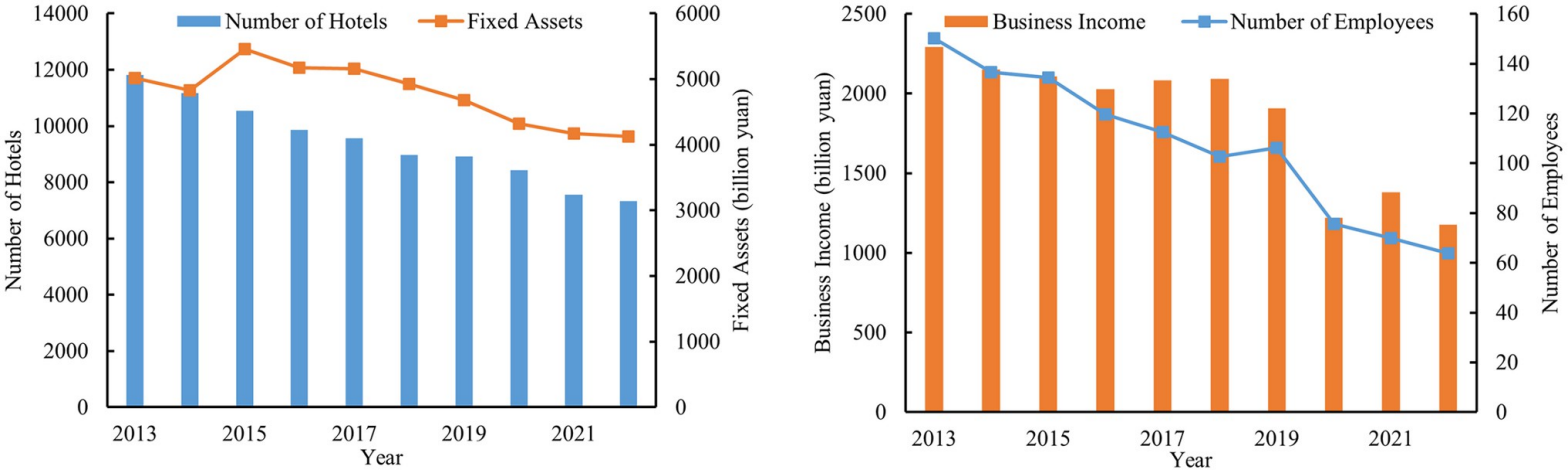
Trends in input and output indicators.

From 2013 to 2022, the development of the star-rated hotel industry in China showed a trend of contraction (Fig 2). In 2013, there were a total of 11,687 star-rated hotels in China, with the total original value of fixed assets amounting to 501.77 billion yuan, the number of employees reached 1.52 million, and the total business income was 229.29 billion yuan. Comparison to 2013, the number of star-rated hotels in China decreased by 37.2% in 2022, standing at 7,337 hotels. The original value of fixed assets, the employees, and the business income were 412.7 billion yuan, 636,900 individuals, and 117.768 billion yuan, respectively, with reductions of 17.8%, 57.6%, and 48.6% respectively. There are significant regional disparities in the development of the star-rated hotel industry across China, with a higher number of star-rated hotels in Beijing, Guangdong, and Zhejiang, and a lower number in the Tibet, Qinghai, and Ningxia.

## 3. Results

### 3.1. The evolution of regional differences in operational efficiency

**Fig 3:**
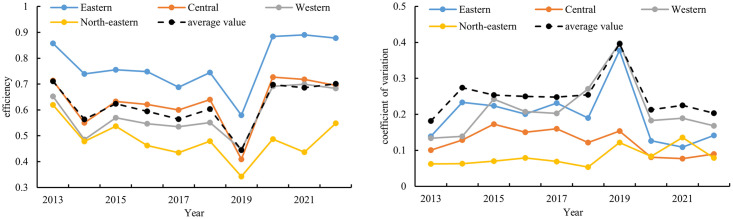
Operational efficiency and coefficient of variation of star-rated hotels.

Based on regulations from the National Bureau of Statistics of China, this paper categorizes the regional attribution of operational efficiency of star-rated hotels in provinces, municipalities, and autonomous regions into Eastern, Central, Western, and Northeastern regions. It calculates the coefficient of variation of operational efficiency for both nationwide and regional star-rated hotels, exploring the degree of internal efficiency differences within each region (Fig 3). During the study period, the overall operational efficiency of star-rated hotels in China exhibited fluctuating characteristics, with the mean value generally ranging between 0.4 and 0.7. Spatially, the operational efficiency of star-rated hotels follows a distinct distribution pattern of " Eastern > Central > Western > Northeastern" regions. The economic development level in the eastern region of China is generally higher. The star-rated hotel industry started early, with a large market demand and extensive operational management experience. The format development is relatively mature, and overall efficiency values typically range between 0.6 and 0.9, significantly higher than the national average efficiency. The operational efficiency values of star-rated hotels in the central and western regions are close to the national average efficiency. Although the overall economic development level in these regions is lower than that in the eastern region, the continuous advancement of the Central Rise Strategy and the Western Development Strategy has fostered a favorable development trend. Industries such as tourism are growing rapidly, providing opportunities for the development of star-rated hotels in these regions. The Northeastern region of China is traditionally an industrial and agricultural base with a single industry structure. The development of tourism reception industries such as the star-rated hotel industry has been relatively lagging, resulting in efficiency values lower than the national average. However, it is currently actively optimizing and adjusting its industrial structure, vigorously promoting ice and snow tourism. By the end of the study period, it showed an upward trend.

From the perspective of the coefficient of variation, there is a significant disparity in the operational efficiency of star-rated hotels among provinces, municipalities, and autonomous regions nationwide. The differences within the Eastern, Central, Western, and Northeastern regions are relatively smaller among their respective provinces. Specifically, within the Eastern and Western regions, there is a greater disparity in efficiency among provinces compared to the Central and Northeastern regions. This indicates a phenomenon of polarization in the development of star-rated hotels between provinces within the Eastern and Western regions, with larger gaps between provinces.

### 3.2. Spatial correlation network analysis of operational efficiency

#### 3.2.1. Overall network characteristics

**Fig 4:**
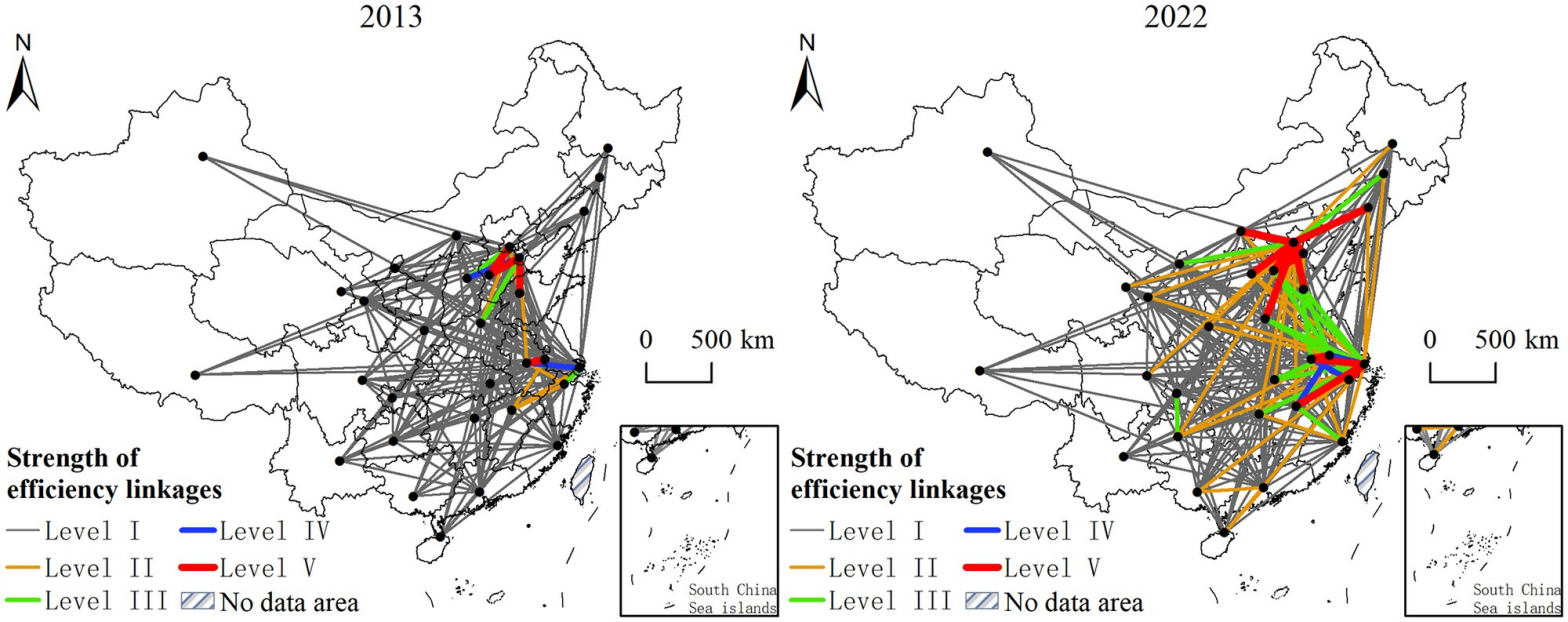
Spatial correlation network of operating efficiency of star-rated hotels.

Using the modified gravity model to calculate the inter-provincial operational efficiency correlation strength of star-rated hotels in China, and employing ArcGIS 10.2 software, combining binary matrices to draw spatial correlation network maps of star-rated hotel operational efficiency in China for the years 2013 and 2022. To facilitate data analysis, the provincial capital cities represent the nodes in the network, and the strength of interprovincial connections is categorized into five levels from lowest to highest (Fig 4). The operational efficiency of star-rated hotels among provinces in China has formed a complex spatial network structure. Non-adjacent provinces have transcended traditional geographical constraints, leading to cross-regional linkage effects, characterized by a "core-edge" spatial structure with Beijing, Tianjin, Shanghai, Jiangsu, Zhejiang, and others as core points. In comparison to 2013, the number of associations among members in the network increased from 205 to 239 by 2022. The overall association strength between members in the eastern and central regions notably improved, showing characteristics of densification, stratification, and strengthening. This improvement is attributed to China’s rapid economic development over the past decade and increasingly convenient transportation services, which have enhanced the breadth and depth of regional cooperation. However, the increase in association strength between provinces in the central and western regions is not as significant, indicating the need for further policy guidance and enhanced inter-regional communication and cooperation.

**Fig 5:**
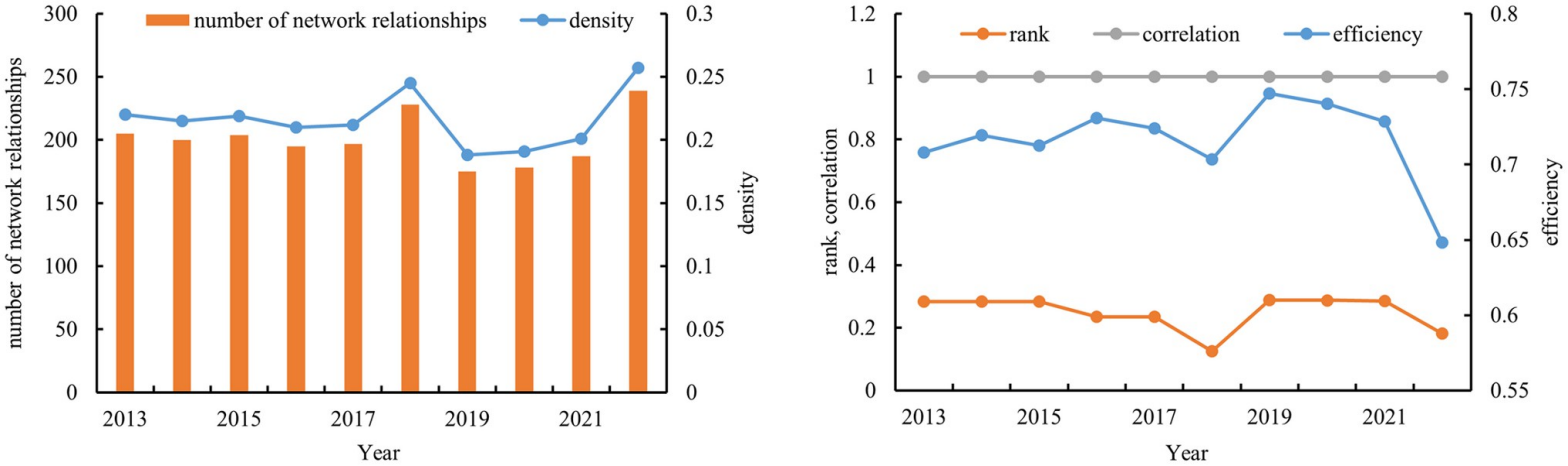
Overall network characteristics of operating efficiency of star-rated hotels.

Constructing the spatial correlation matrix of provincial-level star-rated hotel operational efficiency in China, and using UCINET software to calculate network measures such as network relationships, correlation, network density, network rank, and network efficiency indicators (Fig 5). The trends in network density and network relations of star-rated hotel operational efficiency from 2013 to 2022 show a consistent pattern of "rise-fall-rise." The network density generally remains between 0.2 and 0.3, peaking at 0.257 in 2022 and reaching a minimum of 0.188 in 2019. This indicates that there is a certain level of correlation among provincial-level star-rated hotel operational efficiencies in China, but the network density is relatively low and shows periodic dynamic changes. There is significant room for improvement and optimization. During the study period, the network correlation of star-rated hotel operational efficiency consistently remained at 1, indicating that the overall network structure exhibits good connectivity, accessibility, and robustness. All provinces, municipalities, and autonomous regions are interconnected within the spatial correlation network. The network degree rank ranges from 0.18 to 0.29, indicating that there is no significant spatial hierarchy in star-rated hotel operational efficiency among provinces. This suggests that factors such as personnel and funds flow reasonably between different regions, optimizing resource allocation. The average network efficiency is 0.72, which is moderately high, indicating that the spatial correlation network exhibits strong stability with several overflow channels. However, by the end of the study period, there was a downward trend.

#### 3.2.2. Individual network characteristics

**Fig 6:**
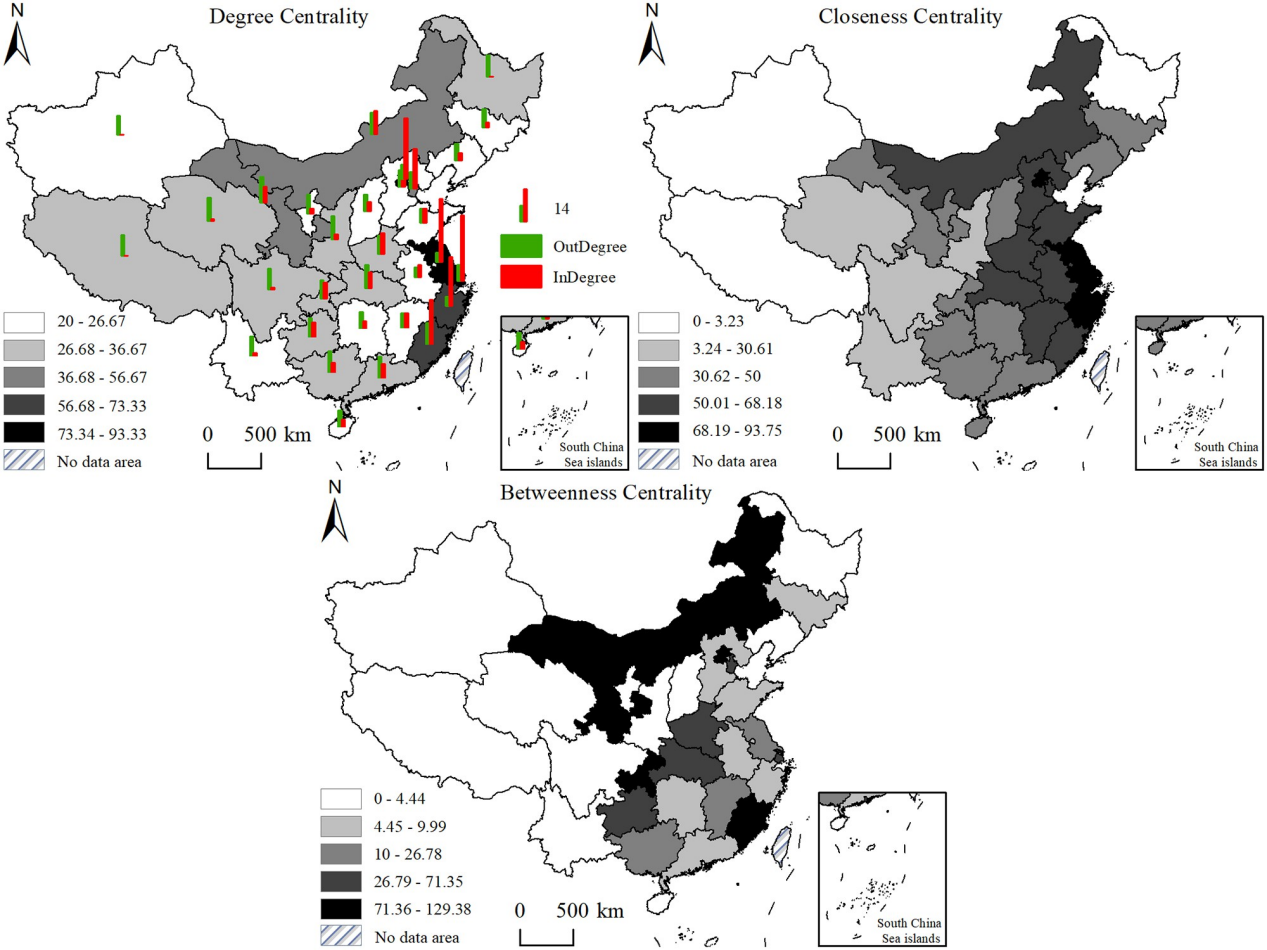
The spatial distribution of degree centrality, closeness centrality, and betweenness centrality in 2022.

Based on the calculation of centrality indicators for all nodes of star-rated hotel operational efficiency in China from 2013 to 2022, it was found that the centrality values of nodes did not show significant differences across all years. Therefore, the centrality analysis for 2022 reveals the individual characteristics of the spatial network structure (Fig 6).

*(1) Degree centrality*. Degree centrality measures the centrality of a node in a network based on the number of connections it has with other nodes [43]. In terms of in-degree and out-degree, Beijing, Tianjin, Shanghai, Jiangsu, and Zhejiang provinces exhibit significantly higher degrees of centrality compared to other provinces in the spatial network of star-rated hotel operational efficiency. They hold core positions and control priority in the network. These provinces are located in the economically developed eastern coastal areas. They benefit from superior geographical location, convenient transportation, high population density, and high consumer spending levels. They have a large demand for star-rated hotel services, which attracts labor and investment from other provinces. In the spatial correlation network of star-rated hotel operational efficiency, they act as recipients of resource overflow. The out-degree centrality of each node is greater than 0, indicating that star-rated hotel operational efficiency in all provinces across the country exhibits spatial radiation effects. Provinces with higher out-degree centrality include Gansu, Shaanxi, Qinghai, and Hubei, which are located in the central and western regions where economic development is relatively slower and geographical advantages are less prominent. They show strong spillover effects in the spatial network.

*(2) Closeness centrality*. Closeness centrality measures the centrality of a node in a network by calculating the average distance between the node and all other nodes it is connected to [44]. The distribution of closeness centrality in China’s star-rated hotel operational efficiency network is relatively balanced, with small inter-provincial differences. Most values fall within the range of 40 to 60, indicating high overall spatial network efficiency in terms of flow. The provinces with the highest closeness centrality are Beijing, Jiangsu, and Shanghai. These provinces have significant advantages in policy frameworks, economic development, technology and culture, and infrastructure. They serve as centers for technology innovation, international exchanges, and economic development in China, playing the role of "central actors" in the spatial network of star-rated hotel operational efficiency. The provinces with the lowest closeness centrality are Heilongjiang, Tibet, and Xinjiang. These provinces have relatively weaker capabilities in accessing technology, information, capital, and human resources. They play a "passive" role in the spatial network.

*(3) Betweenness centrality*. Betweenness centrality is used to represent the "bridge" role of a node in a network, indicating the node’s intermediacy in relationships between other nodes [45]. Provinces with high betweenness centrality include Beijing, Fujian, Inner Mongolia, and Gansu. This indicates that these provinces play a strong "bridge" and "connector" role in the transmission of operational elements and resources among star-rated hotels. Other provinces heavily rely on the interdependencies of operational efficiency relationships facilitated by these provinces. However, provinces such as Heilongjiang, Tibet, Qinghai, and Xinjiang, due to their remote geographical locations, low population density, and relatively underdeveloped economic status, have a betweenness centrality of 0. They difficult to serve as intermediaries to transmit or control the operational efficiency connections of hotels in other provinces.

#### 3.2.3. Spatial clustering structure characteristics

To delve deeper into the roles of nodes in the network of star-rated hotel operational efficiency, using 2022 as an example, the 31 provinces were categorized into three types of clusters using the CONCOR module in UCINET software (Table 3). In 2022, there are a total of 239 relationships in the network of star-rated hotel operational efficiency. Among these, 55 relationships (23.01%) exist within clusters, while 184 relationships (76.99%) exist between plates. This indicates that internal relationships within the network are relatively weak, highlighting significant spillover effects. Plate I expects an internal relationship proportion of 6.67%, but the actual internal relationship proportion is 22.73%. Plate II expects an internal relationship proportion of 10%, but the actual internal relationship proportion is 25%. Both plates have actual internal relationship proportions higher than expected, with more received relationships than spillover relationships, qualifying them as " Net benefit " plates. The members within these plates include Beijing, Tianjin, Jiangsu, Shanghai, and Zhejiang et al. Plate III expects an internal relationship proportion of 46.67%, but the actual internal relationship proportion is 16.80%. The number of spillover relationships far exceeds the received relationships, categorizing it as a "Net overflow " plate. Internal members include provinces from the Northeast and Western regions, such as Heilongjiang, Hebei, Jilin, Liaoning, Tibet Autonomous Region, Gansu, and Qinghai et al. Plate IV expects an internal relationship proportion of 26.67%, which is lower than the actual internal relationship proportion of 33.82%. This plate receives numerous relationships from other provinces and also has substantial spillover relationships, qualifying it as a " Two-way spillover " plate. Members within this plate include Anhui, Guangdong, and Guangxi et al. In various relationship plates, net overflow and two-way spillover plates account for a majority proportion of 77.42%. This indicates that in spatial association networks, most members prioritize factor output. However, provinces with higher efficiency in managing star-rated hotels exhibit very limited spillover effects, characterized by a significant concentration of mobile factor resources towards a few provinces.

**Table 3:** Division of spatial correlation plates of operational efficiency of star-rated hotels.

Plate	Number of received relationships		Number of sent relationships		Expected internal relationship ratio	Actual internal relationship ratio	Plate type
	Inside	Outside	Inside	Outside			
Plate I	5	48	5	17	6.67%	22.73%	Net benefit
Plate II	6	89	6	18	10.00%	25.00%	Net benefit
Plate III	21	23	21	104	46.67%	16.80%	Net overflow
Plate IV	23	24	23	45	26.67%	33.82%	Two-way spillover

**Fig 7:**
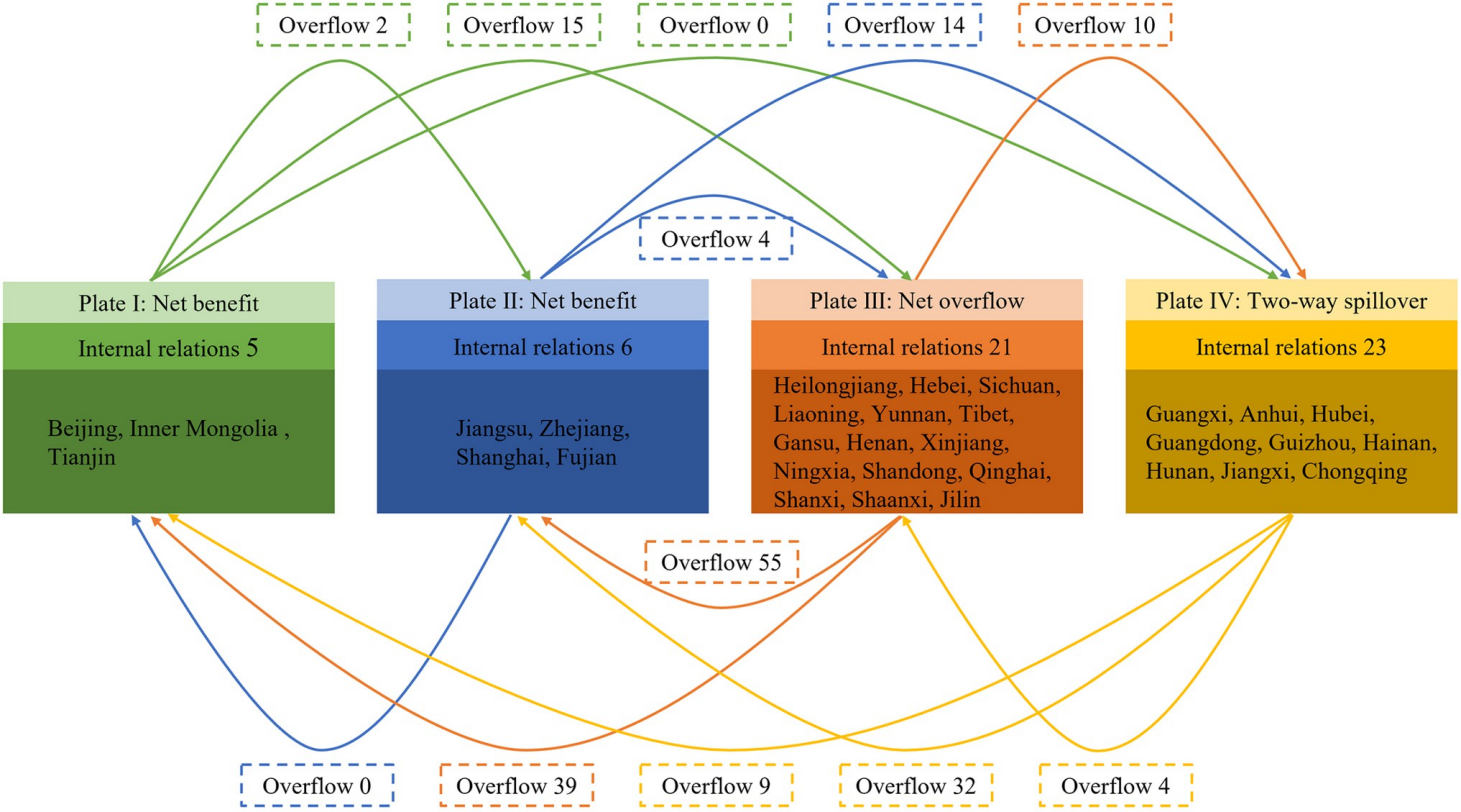
Composition and interaction of network plate members.

By calculating the network density matrices and image matrices of the efficiency of managing star-rated hotels (Table 4), and constructing an interactive communication diagram between plates (Fig 7), we further explore the transmission mechanisms between plates. Arrows in the figure indicate the direction of spillover relationships between plates. In 2022, the overall network density of the efficiency in managing star-rated hotels was 0.257. Using this overall network density as a threshold, the density of relationships between the four plates is binarized: sectors with a density greater than or equal to the overall network density of 0.257 are assigned a value of 1, while those with a density less than 0.257 are assigned a value of 0, thus converting the density matrix into an image matrix. Based on the matrix and interaction diagram, it is evident that plates I and II have good internal relationships, but they have relatively few members. In contrast, plate III and plate IV have more members, but their internal relationships are poor. This results in weaker overall internal network relationships but stronger overflow relationships. Plates I and II consist mainly of provinces with economic prosperity and advantageous geographic locations. Leveraging their favorable conditions, they receive overflow relationships from plate III and IV and within their respective sectors. Plate III is primarily composed of underdeveloped provinces in the northeast and west, with external overflow relationships far exceeding its incoming relationships. Plate IV consists mainly of provinces in the east and central regions, leveraging its strategic location to both receive and emit relationships, with a higher number of internal connections.

**Table 4:** Density matrix and Image matrix of spatial correlation network segments of operational efficiency of star-rated hotels.

Plate	Density matrix				Image matrix			
	Plate I	Plate II	Plate III	Plate IV	Plate I	Plate II	Plate III	Plate IV
Plate I	0.833	0.167	0.333	0	1	0	1	0
Plate II	0	0.5	0.067	0.389	0	1	0	1
Plate III	0.867	0.917	0.1	0.074	1	1	0	0
Plate IV	0.333	0.889	0.03	0.319	1	1	0	1

## 4. Analysis of driving factors of the spatial correlation network

### 4.1. Description of variables

Per capita GDP (PGDP) represents the level of regional economic development. Regions with higher economic development have well-developed infrastructure and optimized industrial structures, leading to a higher level of hospitality industry development, which in turn affects the operational efficiency of star-rated hotels [46]. Per capita consumption level (PCL), this indicator reflects the local residents’ purchasing power for star-rated hotel services and products. Regions with higher PCL have higher living standards and greater demand for high-standard banquets [47]. Permanent resident population (PRP), this indicator reflects the size of the customer base for star-rated hotels [48]. Additionally, the operation and development of star-rated hotels also require support from human resources. Total import and export volume (TIEV), this indicator reflects the level of openness of a region to the outside world [49]. Regions with high levels of openness often host numerous business activities such as conferences and exhibitions, thereby generating demand for star-rated hotels. Spatial distance between provincial capital cities (SDCC) affects the flow of operational elements and competitive relationships among adjacent regions [50], thereby influencing the spatial network morphology of operational efficiency for star-rated hotels. Passenger transport volume (PTV), this indicator represents the region’s transportation hub capacity. Hub capacity affects the accessibility and customer flow market of star-rated hotels [51]. Tourist arrivals (TA) reflect the level of tourism industry development. Regions with a well-developed tourism industry have a high demand for tourist accommodations, thereby influencing the operational efficiency of star-rated hotels [52]. This article selects the difference matrix of the seven aforementioned factors across various regions as the independent variables, and the spatial correlation matrix of star-rated hotel operational efficiency as the dependent variable. To avoid the influence of measurement units on calculation accuracy, the relevant variable matrices are standardized before conducting QAP analysis.

### 4.2. QAP analysis results

The study selected data from four distinct years: 2013, 2016, 2019, and 2022, to thoroughly analyze the impact of various influencing factors on the spatial network distribution of the operational efficiency of star-rated hotels (Table 5). The difference matrix of PGDP and SDCC exhibits a highly stable and significant influence on the operational efficiency of star-rated hotels. This relationship has consistently passed the 5% significance test across the four years under investigation. Specifically, the regression coefficients for the PGDP difference matrix are positive, while the regression coefficient for the SDCC difference matrix is negative. Capital plays a leading role in the production and cooperation of star-rated hotels across different regions. The greater the difference in economic development levels, the more advantageous it is for economically developed regions with well-established infrastructure to attract capital, talent, and other production factors. This process then affects the spatial network correlation strength of operational efficiency for star-rated hotels through "spillover effects" and "trickle-down effects" interacting with less developed provinces. Moreover, increasing geographical distance raises the cost of flow for hotel operational resources between provinces. Therefore, provinces that are closer in distance have a greater likelihood of correlated production and operational relationships among hotels.

**Table 5:** Results of QAP regression analyses.

Variable of Difference matrix	2013		2016		2019		2022	
	coefficient	P-value	coefficient	P-value	coefficient	P-value	coefficient	P-value
PGDP	0.530	0.000	0.511	0.000	0.551	0.000	0.471	0.001
PCL	0.083	0.161	0.170	0.012	0.009	0.461	0.099	0.025
PRP	-0.054	0.119	-0.063	0.058	-0.052	0.126	0.036	0.498
TIEV	0.195	0.026	0.077	0.155	0.258	0.001	-0.127	0.111
SDCC	-0.384	0.000	-0.341	0.000	-0.288	0.000	-0.297	0.002
PTV	0.012	0.365	-0.001	0.503	-0.024	0.274	-0.101	0.322
TA	-0.152	0.052	-0.076	0.127	-0.115	0.027	0.023	0.229

The analysis reveals that the difference matrices of PCL, TIEV, and TA only pass the significance level test in some years, indicating that the impact of these factors on the spatial network of the operational efficiency of star-rated hotels is not very stable. Moreover, the regression coefficients in the years that passed the significance level test are relatively small, suggesting a weaker influence. The local population’s consumption opportunities in star-rated hotels are primarily focused on ceremonial banquets, such as wedding feasts and birthday celebrations. In areas with higher resident consumption levels, the requirements for banquets are relatively high, creating a certain demand for high-star hotels. However, this market demand is not stable. Furthermore, the hotel service industry is characterized by the simultaneity of production and consumption. As the system of external opening matures, the significant proportion of online e-commerce transactions does not fully reflect the demand for hotel services. Currently, the hospitality industry is experiencing fierce competition. The brand appeal of star-rated hotels to tourists has weakened, and the consumer market for star-rated hotels is being significantly encroached upon by alternative accommodations such as homestays and budget chain hotels. These factors may contribute to the stable influence of the PCL, TIEV, and TA difference matrices on the spatial network of operational efficiency in star-rated hotels.

The difference matrices of PRP and PTV exhibit a weak influence on the spatial connectivity of the operational efficiency of star-rated hotels, failing to pass the significance level test in all four years. The number of local residents and transportation capacity can only reflect the potential of the star-rated hotel consumption market, but actual consumption behavior also needs to be considered in conjunction with the market’s purchasing power. Moreover, with the improvement of China’s transportation system, travel efficiency has increased, leading to a significant reduction in transit passengers. These factors may contribute to the inconspicuous impact of the PRP and PTV difference matrices on the spatial connectivity of operational efficiency in star-rated hotels.

## 5. Conclusion and discussion

### 5.1. Conclusion

This article constructed an evaluation index system for the operational efficiency of star-rated hotels and utilized the Super-DEA model to measure the operational efficiency of star-rated hotels in China from 2013 to 2022. By integrating the modified gravity model and social network analysis methods, it analyzed the characteristics and influencing factors of the spatial correlation network structure of operational efficiency among star-rated hotels in China. The study holds significant theoretical and practical implications for the development of the star-rated hotel industry. In terms of theoretical contributions, the research explores the spatial correlation of operational efficiency in star-rated hotels from a network perspective, compensating for the oversight of spatial correlation in previous studies on hotel operational efficiency. This approach broadens the research horizon and enriches the outcomes in the field of hotel operational efficiency research. Regarding practical contributions, the study proposes strategies for enhancing the operational efficiency of star-rated hotels in practice, strengthening regional industrial collaboration, and optimizing spatial network structures. These strategies will be beneficial for promoting the development of the star-rated hotel industry in China and provide a reference for establishing coordinated development mechanisms in the hotel industry across different regions. The research findings are as follows:

1. During the study period, the overall operational efficiency of star-rated hotels in China exhibited fluctuating characteristics. Spatially, the operational efficiency of star-rated hotels showed a distinct distribution pattern of "Eastern > Central > Western > Northeastern". There were varying degrees of operational efficiency differences among star-rated hotels within each region.
2. From overall network characteristics, within the spatial network structure of operational efficiency among star-rated hotels in China, non-adjacent provinces have surpassed traditional geographic constraints to create cross-regional linkage effects. The network as a whole exhibits’ characteristics of densification, hierarchy, and reinforcement. However, the overall network density is relatively low, indicating significant potential for improvement and optimization. From the perspective of individual network characteristics, eastern provinces such as Beijing, Shanghai, and Jiangsu play pivotal roles as "central actors" in the spatial network of operational efficiency among star-rated hotels. Provinces like Beijing, Fujian, Inner Mongolia, and Gansu serve as robust "bridges" and "links" within the spatial network. Northeastern provinces like Heilongjiang, Tibet, and Xinjiang, as well as some western and parts of central regions, have less tight connections with other provinces, thus playing more passive roles in the spatial network.
3. The block model results indicate that the members of the net benefit plate and the Two-way spillover plate are mainly from eastern and some central provinces. The net overflow plate members are primarily from northeastern and western provinces. In the spatial correlation network of operational efficiency among star-rated hotels in China, most members emphasize the output of resources, characterized by a significant concentration of flow elements towards a few provinces.
4. The QAP analysis results indicate that the differences in economic development levels and the distances to provincial capital cities have a significant and stable impact on the correlation strength of the operational efficiency of star-rated hotels, and are key factors influencing the spatial network structure of the operational efficiency of star-rated hotels in Chinese provincial regions. In contrast, the differences in resident consumption levels, regional population sizes, levels of openness to the outside world, transportation capabilities, and levels of tourism industry development have a weaker influence on the spatial connections of the operational efficiency of star-rated hotels.

### 5.2. Discussion

Based on the above research findings, to further enhance the operational efficiency of star-rated hotels in China and optimize the spatial network structure of operational efficiency, this article proposes the following policy recommendations:

1. From the perspective of the operational efficiency of star-rated hotels in each region, government departments should formulate development policies and quality standards documents that are aligned with the needs of star-rated hotel development. It is important to enhance guidance and supervision mechanisms to establish the credibility of star-rated hotel brands. Each star-rated hotel should establish a service philosophy centered around customer experience, emphasizing the quality of both hardware facilities and software services. It is also essential to cultivate a distinctive corporate culture to enhance employee and customer loyalty toward the star-rated hotel. This approach aims to enhance the industry competitiveness of star-rated hotels, strengthen their ability to withstand operational risks, and consistently improve operational efficiency.
2. From overall and individual spatial network characteristics of operational efficiency of star-rated hotels, it is necessary to fully leverage the functional roles of government departments and organizations such as the National Tourism and Hotel Association. Establishing and improving regional cooperation mechanisms for star-rated hotels is crucial. This includes forming strategic alliances among star-rated hotel accommodation brands to facilitate mutual exchange of guests and information across regions. This initiative aims to strengthen the spatial interconnections of operational efficiency among star-rated hotels across provinces.
3. From the block model analysis, it is necessary to break through the club convergence effect of operational efficiency among star-rated hotels across various plates. This involves enhancing bidirectional spillover effects between plates and strengthening their interrelationships. It is crucial to fully leverage the radiating effect of net benefit plate members such as Beijing, Tianjin, Shanghai, Jiangsu, and Zhejiang to facilitate the orderly outflow of capital, markets, and talent. Northeastern and central-western regions should focus on improving infrastructure such as transportation and enhancing policies for talent attraction and investment promotion. This approach aims to elevate the development level of their star-rated hotel industry and reduce their spillover effects.
4. From the QAP analysis, each region should leverage its advantageous resources to optimize industrial structure, enhance regional economic development, improve people’s living standards, increase demand for star-rated hotels, and boost revenue. Simultaneously, efforts should be accelerated in constructing transportation infrastructure and innovating in information technology. This will harness the spatiotemporal compression effects of transportation and information services, thereby fostering closer connections among provinces in the spatial network of operational efficiency for star-rated hotels.

This study analyzed the evolution characteristics and influencing factors of the spatial correlation network structure of operational efficiency among star-rated hotels in China from complex networks. It provides a new perspective for research on the development of star-rated hotels from a geographical perspective. However, the study still has the following limitations: Due to the accessibility of data, the study chose to analyze the network structure of operational efficiency among star-rated hotels at the provincial level. As a result, the findings may lack precision in terms of research scale. The study chose spatial distance as the measure between provinces. With the development of transportation networks, the impact of spatial distance on the spatial network of operational efficiency among star-rated hotels is gradually weakening. Therefore, time distance might be more appropriate as a measure. The selection of influencing factors in the spatial network of operational efficiency among star-rated hotels may not be comprehensive enough, as it could also be influenced by policies and geographical factors. Future research could further improve by refining the research scale, selecting indicators, and enhancing the study results.
